# Improvement in Mechanical Properties of Completely Decomposed Granite Soil Concrete Fabricated with Pre-Setting Pressurization

**DOI:** 10.3390/ma17174314

**Published:** 2024-08-30

**Authors:** Yi Song, Zhongqi Quentin Yue, Yanlu Ding

**Affiliations:** 1 Department of Civil Engineering, China University of Geosciences (Beijing), Beijing 100083, China; songyi@cugb.edu.cn; 2 School of Science, Harbin Institute of Technology, Shenzhen 518057, China; 3 Formerly Department of Civil Engineering, The University of Hong Kong, Hong Kong, China; 4 Key Laboratory of Urban Security and Disaster Engineering of China Ministry of Education, Beijing University of Technology, Beijing 100021, China; ylding@bjut.edu.cn

**Keywords:** pre-setting pressurization, completely decomposed granite, concrete, mechanical properties, compacted density, uniaxial compressive strength, porosity

## Abstract

This paper investigates the effectiveness of applying continuous high-compression pressure on the initial setting of fresh concrete to produce hardened concrete materials with excellent mechanical properties. A novel experimental apparatus was self-designed and used for the pre-setting pressure application. The utilization of the completely decomposed granite (CDG) soil as an alternative aggregate in concrete production was also explored. A total of twenty-eight specimens were fabricated using two types of fine aggregates, six mix ratios, two initial pressure values, and two distinct durations of the initial pressure application. The density and uniaxial compressive strength (UCS) of the specimens were examined to evaluate their mechanical qualities, while micro-CT tests with image analysis were used to quantify their porosity. The results indicated that the 10 MPa initial pre-setting pressurization can effectively eliminate the excess air and voids within the fresh concrete, therefore enhancing the mechanical properties of the hardened concrete specimens of various types. Compared with non-pressurized specimens, the porosity values of pressurized specimens were reduced by 73.11% to 86.53%, the density values were increased by 1.43% to 8.31%, and the UCS values were increased by 8.42% to 187.43%. These findings provide a reference for using a continuous high pre-setting compression pressure and using CDG soil as an aggregate in the fabrication of concrete materials with improved mechanical performance.

## 1. Introduction

Portland cement concrete is a composite material obtained by an appropriate proportioned mixture of aggregate, Portland cement, water, and other additives [1,2,3]. Concrete materials have been the second most consumed material in the world for many years due to the rapid urbanization and industrialization [1,2]. Initially, the fresh fluid mixture has a hydration reaction, and then gradually hardens into the solid material with high elastic modulus and compressive and tensile strength [3,4]. The pore properties, including pore structures, pore distribution, and porosity, of hardened concrete materials have significant influence on its mechanical performance. For instance, it has been demonstrated that the increase in porosity of concrete can result in the decline of its compressive strength [3,5,6]. Therefore, various approaches, including expelling excess water and applying external mechanical pressure to fresh cement paste to reduce the porosity and improve the mechanical properties of hardened cement paste, have been thoroughly investigated.

Researchers have attempted to apply high-compaction pressure to fresh concrete mixtures in the liquid state since the 1970s to obtain high-performance concrete [7]. It has been generally demonstrated that the pre-setting pressurization can effectively enhance the hydration efficiency of cement and improve the mechanical properties, including the compressive strength and tensile strength of hardened concrete [8,9,10,11,12,13,14,15,16]. Justs et al. [9] investigated the effectiveness of pressurization during the concrete hardening process in the manufacture of ultra-high-performance concrete. Their findings revealed that the 10 MPa initial pressurization could increase the compressive strength of hardened concrete by 31.5%, while a higher initial pressurization of 50 MPa resulted in a 48% increase in the compressive strength. Meanwhile, Nematzadeh et al. [13,14] utilized the compaction technique to expel entrapped air and excess water in the fresh concrete, increasing the compressive strength of hardened concrete by 185.9%. Wang et al. [15,16] and Kazmi et al. [12] developed a new layered compression casting method to improve the performance of normal concrete and concrete with recycled aggregates and found that the compressive strength and elastic modulus of layered compression casting concrete (LCC concrete) were significantly improved, by up to 100% and 50%, respectively. Additionally, the pre-setting pressurization technique can also lead to increased density, enhanced homogeneity, and reduced porosity of concrete materials. Consequently, in this study, a novel self-designed hydraulic system is used to apply pre-setting compaction pressure to fresh concrete during its hardening process, with the aim of enhancing the mechanical properties of hardened concrete without additional additives, chemicals, and extra costs.

On the other hand, river sand and crushed rock, which are cheap and easy to acquire, have been used as conventional aggregates. However, the excessive excavations of these natural resources have resulted in serious environmental problems, such as the non-reversible erosion of riverbeds, the collapse of riverbanks, the reduction of hard rock resources, and an increase in greenhouse gas emissions [17,18,19]. Thus, different substitute sources of aggregates, such as industrial by-products and recycled aggregates, have been investigated in previous research [20,21,22,23].

Completely decomposed granite (CDG) is a type of soil, as the in situ weathering product of granitic rocks. CDG can be reddish-brown, well-graded, silty, and fine gravelly sand with proper angularity. The parent granitic rock of the CDG soil has the four main minerals of quartz (SiO_2_), orthoclase feldspar (KAlSi_3_O_8_), plagioclase feldspar (NaAlSi_3_O_8_-CaAl_2_Si_2_O_8_), and biotite (K(Mg, Fe)_3_(AlSi_3_O_10_)(OH)_2_). In the weathering process, quartz is the most resistant to weathering, followed by feldspar and biotite, which are broken down into clay minerals [23,24]. Consequently, the constitutive particles of CDG soil are dominantly composed of quartz, clay minerals, and a small amount of feldspar, and the main elements of CDG soil are O, Si, Al, K, Fe, Na, Ca, and Mg, which are consistent with the elements in the four main minerals of the parent rocks. Illite accounts for 19% while kaolinite accounts for 21% of the CDG soil [25,26]. CDG is abundant in Hong Kong since about 30% of its total land surface area is occupied by granitic rocks, including granite, granodiorite, and quartz monzonite. The depth of weathering can be 100 m deep from the ground surface [23,24,27,28]. CDG was commonly used as landfill for reclamation in the past. Previous studies have extensively examined the physical and mechanical properties of CDG soil, as well as the methodologies for fabricating geopolymers utilizing CDG soil. The mechanical performance of these geopolymers has also been investigated [29,30]. In this paper, as the main mineral in CDG soil is quartz, which is similar to the components of river sand and crushed granitic rocks, CDG soil is considered to be used as an alternative aggregate in concrete production and, consequently, the feasibility is examined.

In summary, concretes with excellent material properties and low cost have been studied for centuries. Traditional methods for improving the mechanical properties and durability of concrete materials include adding fiber-reinforced concrete (FRC) during concrete casting and pre-stressed concrete. However, these methods present certain limitations, such as a reduction in the workability of fresh concrete, complicated construction procedures, and higher costs [31,32,33,34]. Hence, in this study, an innovative pre-setting pressurization technique is proposed and applied on the fresh concrete mixture to effectively enhance the performance of the concrete material. Meanwhile, CDG soil, which is easily accessible, environmentally friendly, and low cost, is considered as a raw material for aggregates in the manufacture of concrete in this study. Overall, this paper explores the potential of utilizing pre-setting pressurization to improve the mechanical performance of concrete specimens using CDG soil as a substitute aggregate. Findings from this investigation provide innovative approaches in the production industries of environmentally friendly concrete materials with low cost and enhanced properties.

## 2. Materials and Methods

### 2.1. Materials

The Portland cement used in the test had the strength class of 52.5 N.

**Figure 1:**
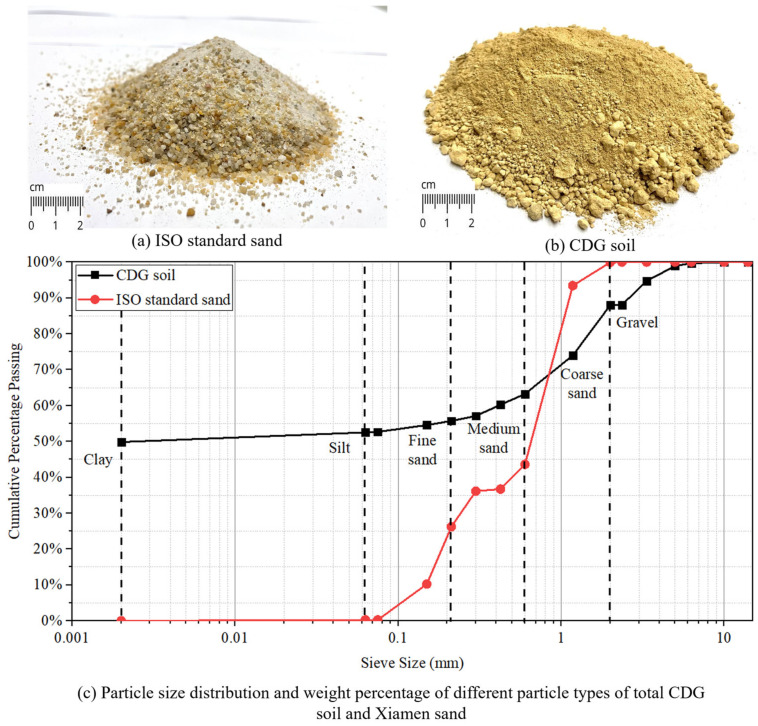
The CDG soil and sand for fine aggregates for fabrication of concrete: (**a**) image of Xiamen sand, (**b**) image of CDG soil, and (**c**) particle size distribution and weight percentage of different particle types of CDG soil and Xiamen sand.

According to the British standard [35,36], Xiamen ISO standard sand (or Xiamen sand or sand), as shown in Figure 1a, was used to cast conventional concrete specimens as a control group in order to perform comparison and evaluation.

For the experimental group, a local CDG soil without further treatment was used as an aggregate in this research, as shown in Figure 1b. The CDG soil was excavated from a construction site in Happy Valley, Hong Kong Island. The parent rock was the equigranular, medium-grained biotite monzogranite, and the stratigraphy was cretaceous intrusive rocks [24]. After wet sieving and dry sieving, the particle size distribution of the ISO standard sand and the CDG soil was as shown in Table 1. The total gradation curve of CDG soil compared with the gradation of ISO sand is shown in Figure 1c. According to the results and gradation curves, it can be illustrated that compared with ISO standard sand, CDG soil had better gradation and a wider particle size distribution range. Specifically, the ISO standard sand consisted entirely of sand with a particle size of 0.063 to 2.36 mm, while the CDG soil contained up to 49.83% clay particles (particle size < 0.002 mm), 2.69% silt particles (particle size of 0.002–0.063 mm), 35.44% sand particles, and 12.04% gravel particles (particle size > 2.36 mm). Due to the smaller particle sizes and larger specific surface area of the clay component in CDG soil, the water absorption of CDG soil used as aggregates will be significantly higher than that of ISO standard sand. Consequently, the utilization of CDG soil in concrete may lower the hydration efficiency of cement in fresh concrete.

### 2.2. Experimental Apparatus and Procedure

**Figure 2:**
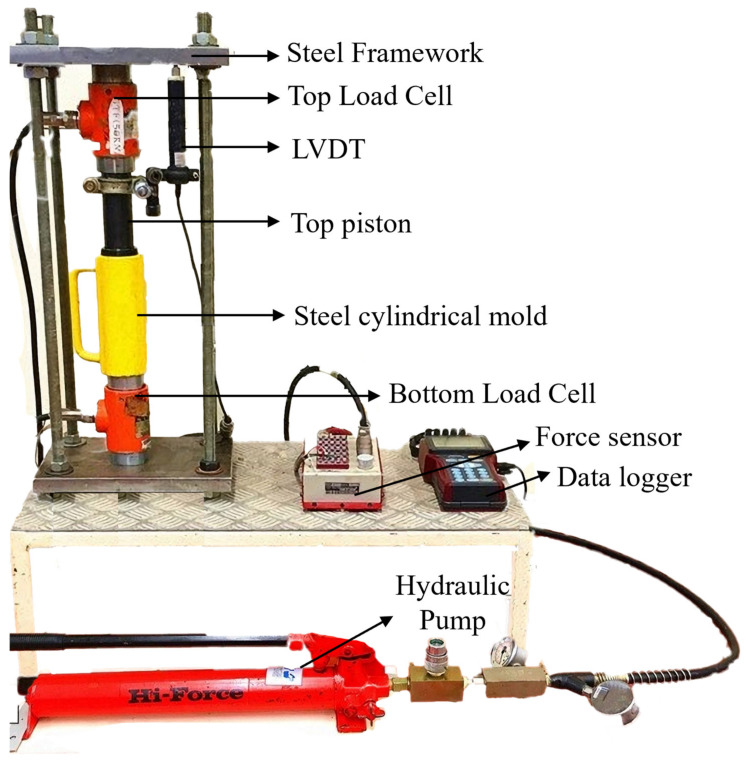
Experimental apparatus for fabrication of the concrete specimens with pre-setting pressurization.

The details of the experimental apparatus for the pressurized concrete fabrication are shown in Figure 2. The apparatus was composed of a hydraulic system, a steel hollow cylinder (50 mm in diameter and 150 mm in height), a top piston with a long rod (50 mm in diameter and 100 mm in height), a bottom piston (50 mm in diameter and 20 mm in height), sealing rubber rings, a force sensor, a data logger, a Linear Variable Differential Transformer (LVDT), and a steel reaction framework.

Upon completion of the preparation of fresh concrete designated for pressurization, the mixture was poured into the steel cylindrical mold equipped with the bottom piston with sealing rubber rings. Subsequently, we assembled the top load cell, top piston, steel mold with fresh concrete, and bottom load cell in a precise sequence within the steel reaction framework. Then, the fabrication of the concrete specimens with pre-setting pressurization could be started.

**Figure 3:**
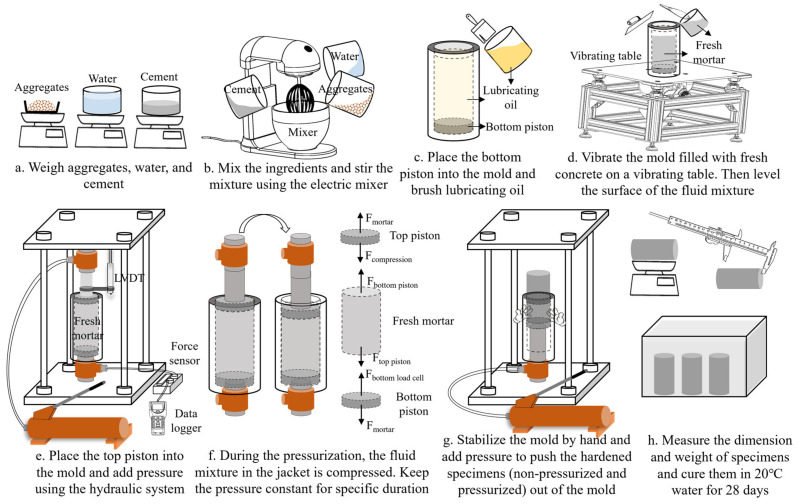
Procedure for casting concrete specimens with 0/10 MPa pre-setting pressurization.

In the application of pre-setting pressure to the initial fresh concrete, the hydraulic pump was employed to facilitate the downward movement of the top load cell via oil pressure. This movement exerted downward pressure on the top piston installed on the steel mold, while the bottom piston was held immovable by the bottom load cell, and the steel framework provided a reactionary force. Correspondingly, the compaction pressure was transmitted to the fresh concrete within the mold. The mechanical process is illustrated in Figure 3f. The induced displacement of the top piston and the applied force during this pressurization process were meticulously captured and recorded by the LVDT, the force sensor, and the data logger, respectively. After a specific pressurization duration, the hardened concrete specimens within the mold could be pushed out using the hydraulic system. More details of the pre-setting pressurization procedure are explained below.

As shown in Figure 3, the casting procedure of pre-setting pressurized concrete specimens can be summarized in the following steps:

(a) Precisely weigh water, cement, and aggregates according to a specific designed mix ratio.

(b) Combine the three components in a steel container and stir the mixture using the electric mixer for 3 min.

(c) Install the bottom piston (50 mm in diameter and 20 mm in height) equipped with a sealing rubber ring into the cylindrical mold, then brush lubricating oil onto the inner surface of the mold in order to facilitate subsequent demolding.

(d) Pour the fluid mixture of fresh concrete into the mold and vibrate the mold with fresh concrete on a vibrating table for about 1 min. Then, level the surface of the fluid mixture in the cylindrical mold.

(e) Install the top piston (50 mm in diameter and 100 mm in height) with the sealing rubber ring into the cylindrical mold, place the mold on the bottom load cell, and attach the top load cell connected to the hydraulic pump on the top piston. Ensuring contact between the top load cell’s surface with the top plate of the steel framework and the bottom load cell’s surface with the bottom plate, proceed to connect the force sensor to the bottom load cell and assemble the LVDT onto the top piston rod.

(f) Apply the compression pressure (10 MPa) to the initial fluid mixture of fresh concrete using the hydraulic system. During the pressurization process, the bottom piston of the mold is held immovable by the bottom load cell, while the top load cell propels the top piston downward, thereby transferring the compression pressure onto the fresh concrete mixture within the mold. Real-time monitoring of the pressure applied on the mixture and the induced downward movement of the top piston is facilitated through the force sensor and LVDT, respectively. Maintain a constant pressure for a specified duration (8 or 24 h).

(g) Upon completion of the designated pressurization duration, unload the pressure, and then the top load cell moves upward correspondingly. Subsequently, remove the top load cell, LVDT, and the force sensor, then reconnect the hydraulic pump to the bottom load cell. Manually hold and stabilize the cylindrical mold and apply pressure using the hydraulic system. With the bottom load cell moving upward and the bottom piston being pushed up, the hardened pressurized concrete specimens can be pushed out of the cylindrical mold.

(h) Measure the dimensions and weight of the pressurized concrete specimens. Cure the hardened specimens in 20 °C water for 28 days and then test them.

For specimens with zero pressurization, fabricated as the control group, after mixing the aggregates, cement, and water of the same ratios as those for pressurized specimens, we poured the fresh concrete into cylindrical molds installed with a bottom piston. The dimensions of the mold and bottom piston were all the same as those for the pressurized specimens (cylindrical mold with a height of 150 mm and diameter of 50 mm, and piston with a height of 20 mm and diameter of 50 mm). Subsequently, we subjected the mold filled with fresh concrete to the vibrating table for 1 min, then leveled the surface of the fluid mixture in the mold. Finally, we rested the mold with fresh concrete for 24 h and then demolded the non-pressurized hardened specimens using the same method as the pressurized specimens (as illustrated in Figure 3g). After 28 days of 20 °C water curing, we tested these specimens.

It is noteworthy that in order to ensure the same water–cement ratios between the pressurized and non-pressurized concrete specimens, both the top piston and the bottom piston of the cylindrical mold were equipped with sealing rubber rings. This ensured that the water within the fresh concrete mixture cannot leak out from the mold due to compression during the pressurization process. Simultaneously, throughout the pressurization duration, as the top piston descended, the fresh concrete was compressed, while the air entrapped within internal pores could be expelled and the voids could be reduced. Consequently, from a macroscopic standpoint, the resulting height measurements of the hardened pressurized concrete specimens were generally lower compared to those of the non-pressurized concrete specimens, potentially introducing a size effect in subsequent uniaxial compressive strength (UCS) tests. In this study, the size effect arising from the differential heights of the specimens was disregarded.

### 2.3. Variation in Axial Force and Displacement with Time during Pre-Setting Pressurization

**Figure 4:**
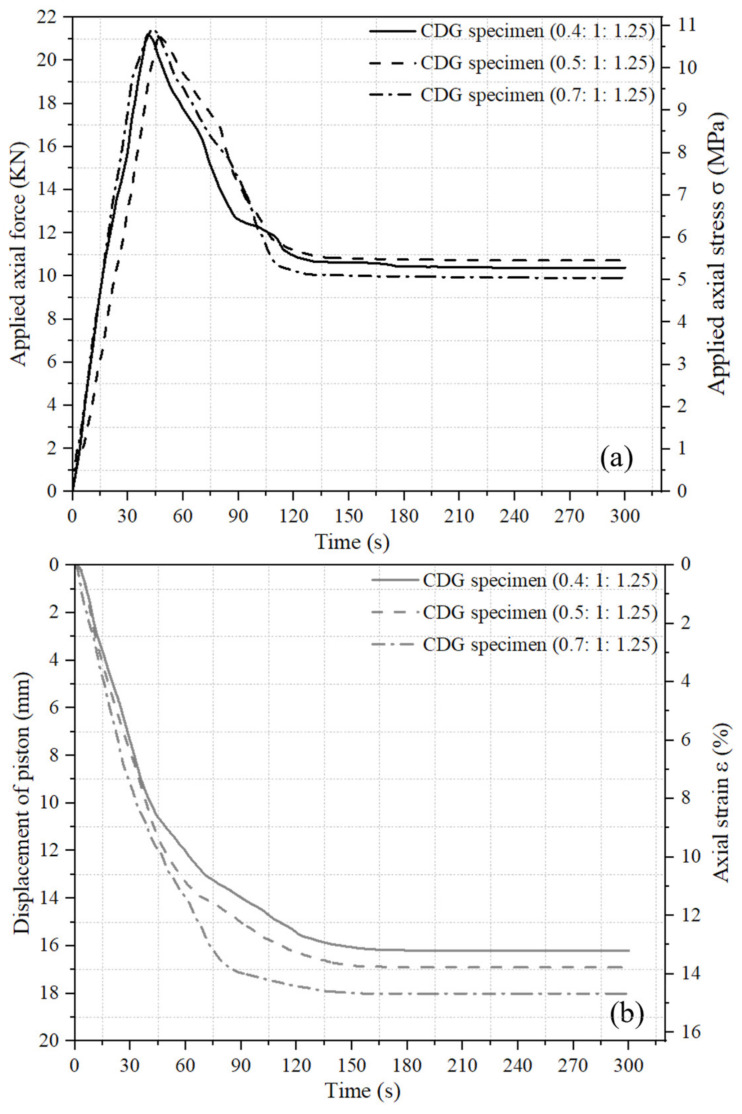
Variations in applied axial force and induced piston displacement with time of CDG concrete specimens: (**a**) applied axial force or stress and (**b**) induced piston displacement or strain.

**Figure 5:**
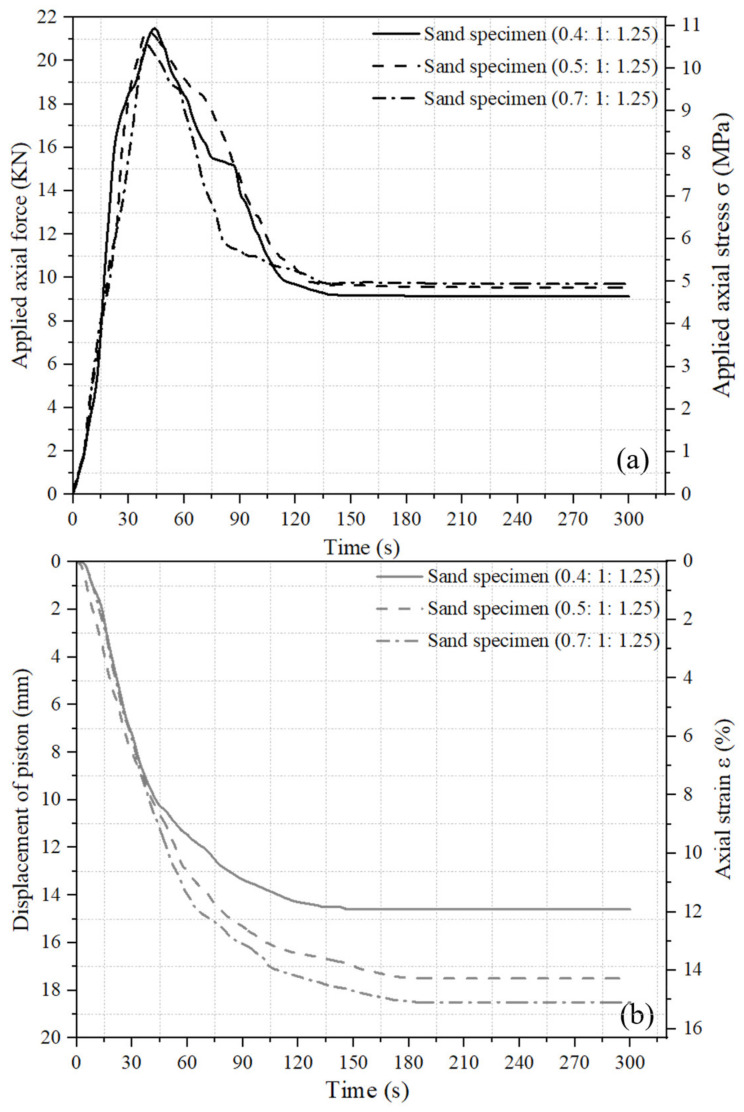
Variations in applied axial force and induced piston displacement with time of sand concrete specimens: (**a**) applied axial force or stress and (**b**) induced piston displacement or strain.

As illustrated above, the variations in the applied axial force and induced displacement of the piston with time during the initial 300 s of pre-setting pressurization were recorded by the force sensor and LVDT, respectively. Curves of concrete specimens using CDG soil and sand as aggregates with three mix ratios (0.4:1:1.25, 0.5:1:1.25, and 0.7:1:1.25) are shown in Figure 4 and Figure 5, respectively. As shown in Figure 4a and Figure 5a, when the pre-setting pressurization was applied using the hydraulic cylinder, the applied axial force firstly rose to 20–22 KN at a roughly linear speed within 40–50 s, which induced the vertical displacement of the piston with time. Then, the increase in the axial force was stopped. At this stopping time, the applied axial force began to drop rapidly as the piston displacement continuously increased, and the hydraulic system could not maintain a constant axial force level. The decreasing speed of the axial force gradually slowed down, and finally stabilized at 9–11 KN at around 80–140 s. For CDG concrete and sand concrete with three types of mix ratios, the trend of axial force variation and the final stable force during the pressurization process were comparable.

As shown in Figure 4b and Figure 5b, once the axial force was applied, the piston began to move downward under the applied force, and the fresh concrete in the cylinder was compressed; then, the piston displaced downward at a near linear rate at first, and then the displacement speed slowed down at 40–50 s after the axial force was applied. Finally, the piston displacement stabilized at 14–19 mm at around 130–180 s. It can be observed from Figure 4 and Figure 5 that as the applied axial force increased linearly, the piston displacement increased linearly too. When the force stopped increasing, the piston displacement continued to increase for another 90–130 s, which caused a decrease in both the applied force and the piton displacement speed. When the piston displacement became stable, the applied force also became stable. For both CDG concrete and sand concrete, as the water–cement ratio (water content) of the specimens increased, the final stable compression height correspondingly increased. This was attributed to the fact that the higher the water content of the fresh concrete, the better the workability and compressibility of it.

### 2.4. Variation in Axial Strain Versus the Applied Axial Stress during Pre-Setting Pressurization

As the inner diameter of the cylinder was 50 mm, the stress of the fresh concrete mixture induced by the applied axial force can be calculated using the following equation:

(1) $$Stress\;\sigma =\frac{Applied\;axial\;force}{{Area}_{cross-section\;of\;cylinder}}=\frac{Applied\;axial\;force}{\pi \times 0.025^{2}}$$

Secondly, as the height of the cylinder was 150 mm, and the height of the piston was 20 mm, the initial height of the fresh concrete mixture in the mold before the pressure was applied can be equal to 130 mm. The axial strain (volumetric strain) of the fresh mixture induced by applied axial force can be calculated using the following equation:

(2) $$Axial\;strain\;\varepsilon =\frac{Displacement\;of\;piston}{Initial\;height\;of\;fresh\;mixture}\times 100\%=\frac{Displacement\;of\;piston}{130}\times 100\%$$

**Figure 6:**
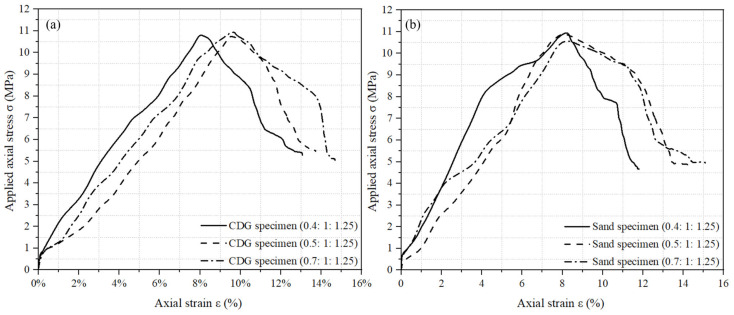
Variation in axial strain (volumetric strain) versus the applied axial stress for the pre-setting pressurization: (**a**) CDG concrete specimens and (**b**) sand concrete specimens.

Figure 6 shows the relationship between axial strain and applied axial stress of CDG concrete and sand concrete specimens with three mix ratios. The stress variation trend for both CDG concrete and sand concrete specimens followed a similar pattern as the applied force over time. The stress value firstly increased almost linearly before reaching the peak value, which was 10–11 MPa. Then, the stress value decreased and finally stabilized at 4.5–5.5 MPa, while the strain stopped at 11–15%.

Since the fresh concrete in the cylinder was in the state of liquid or semi-liquid, Pascal’s principle was applicable so that the piston could transmit the stress to the fresh concrete. Thus, the fresh concrete in the cylinder was firstly subjected to 10–11 MPa pressure, and then subjected to a stable 4.5–5.5 MPa for a specific duration (24 h/8 h).

### 2.5. Prepared Specimens

Referring to the British standard [35], the mixtures were designed according to the following six weight ratios of the water:cement:aggregates, at 0.4:1:1.25, 0.4:1:1, 0.5:1:1.25, 0.5:1:1, 0.7:1:1.25, and 0.7:1:1, respectively. They were used for each of the two aggregate types of CDG soil and sand for the comparative experimental study. For the CDG soil and sand concrete specimens with mix ratios of 0.5:1:1.25 and 0.5:1:1, 8 h pressurization was applied to compare the effectiveness of pressurization with different duration.

**Figure 7:**
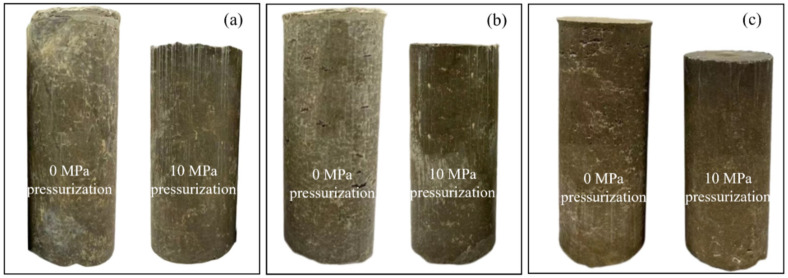
Pictures of six CDG concrete specimens with three typical mix ratios: (**a**) CDG concrete specimens (0.4:1:1.25), (**b**) CDG concrete specimens (0.5:1:1.25), and (**c**) CDG concrete specimens (0.7:1:1.25).

**Figure 8:**
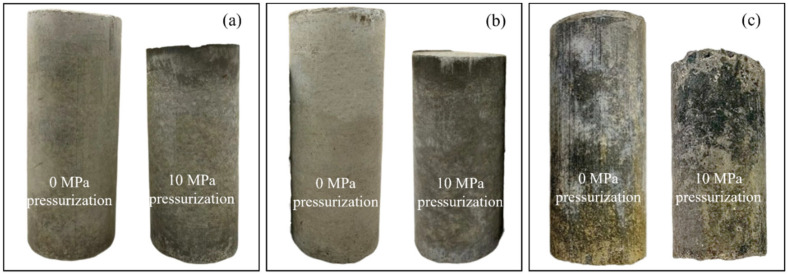
Pictures of six sand concrete specimens with three typical mix ratios: (**a**) sand concrete specimens (0.4:1:1.25), (**b**) sand concrete specimens (0.5:1:1.25), and (**c**) sand concrete specimens (0.7:1:1.25).

Hence, a total of 28 different specimens were fabricated and analyzed, as shown in Table 2. Figure 7 and Figure 8 show pictures of six CDG concrete and six sand concrete specimens with three typical mix ratios.

## 3. Enhanced Properties of Hardened Concrete Specimens

**Figure 9:**
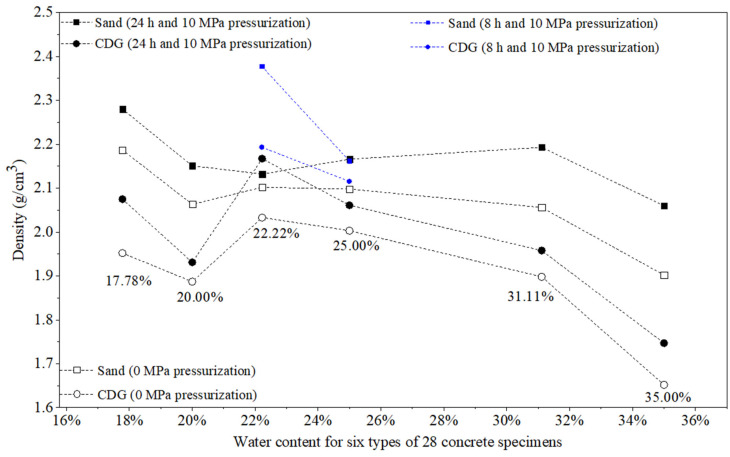
Variations in the density values of 28 concrete specimens fabricated with CDG and Xiamen standard sand under 10 MPa or 0 MPa pre-setting pressurization with different water contents (water mass over mass of cement and aggregate in %).

**Figure 10:**
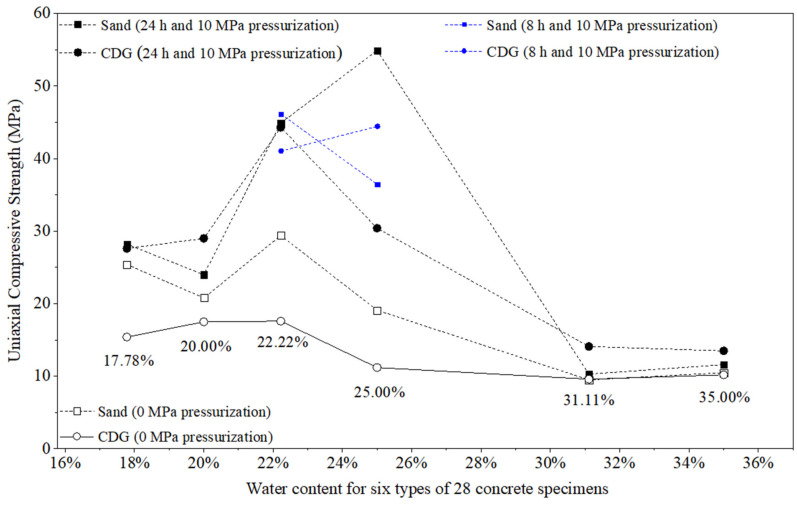
Variations in the UCS values of 28 concrete specimens fabricated with CDG and Xiamen standard sand under 10 MPa or 0 MPa pre-setting pressurization with different water contents (water mass over mass of cement and aggregate in %).

Table 2 and Figure 9 and Figure 10 show the values of the density and compressive strengths (UCS) of the 28 concrete specimens fabricated with the six mixture ratios and with or without the application of 10 MPa pre-setting compression pressure for 24 or 8 h.

### 3.1. Results of Density

#### 3.1.1. Effect of Water–Cement–Aggregate Ratio

In general, the concrete specimens with the water:cement:aggregate ratio of x:1:1.25 had higher density values than those with the water:cement:aggregate ratio of x:1:1, where x = 0.4, 0.5, and 0.7, respectively, which shows that the more aggregates, the higher the density. Furthermore, as x increased from 0.4 to 0.5, and then to 0.7 (the water content (water mass over solid mass) increased from 17.78% to 35.00%), the density values decreased initially and then increased and decreased. Inadequate water content in fresh concrete resulted in reduced hydration efficiency of the cement, whereas an excess of water led to the presence of pores or bubbles within the concrete matrix. Both conditions affected the compactness of the concrete and consequently decreased its overall density. For example, the four CDG specimens (x:1:1.25) with 10 MPa pressurization could have the density values of 2.075, 2.167, 2.193, and 1.958 g/cm^3^ (average = 2.099 g/cm^3^) for x = 0.4, 0.5, and 0.7, respectively. The other four CDG specimens (x:1:1) with 10 MPa pressurization could have the density values of 1.931, 2.061, 2.115, and 1.747 g/cm^3^ (average = 1.964 g/cm^3^) for x = 0.4, 0.5, and 0.7, respectively. The optimum water–cement ratio was 0.5 (the water content was about 22.22% to 25.00%).

#### 3.1.2. Effect of the Pre-Setting Pressurization

The 10 MPa pressurization could significantly increase the density values of the specimens for all six types of mix design. For example, the four CDG specimens (x:1:1.25) with 10 MPa pressurization could have the average density value of 2.099 g/cm^3^. The four CDG specimens (x:1:1.25) with 0 MPa pressurization had the average density value of 1.975 g/cm^3^. For sand concrete specimens with a mix ratio of x:1:1.25, the average density values of pressurized specimens and non-pressurized specimens were 2.246 g/cm^3^ and 2.112 g/cm^3^, respectively. The average increase in CDG concrete and sand concrete specimens under 10 MPa pressurization was 6.3%, which is high. Specifically, CDG concrete and sand concrete specimens hardened under 8 h pressurization exhibited average increases in density values of 6.73% and 8.17%, respectively, which is a little higher than the average increase. Therefore, the 8 h duration of the pre-setting pressurization could have an increasing effect on the density similar to that of the 24 h duration.

#### 3.1.3. Comparison between CDG and Sand

In general, the concrete specimens fabricated with CDG had density values lower than those of the concrete specimens fabricated with sand. However, the CDG concrete specimen (0.5:1:1.25) with 24 h and 10 MPa pressurization had a density (2.167 g/cm^3^) slightly higher than that (2.132 g/cm^3^) of the sand concrete under the same conditions. The CDG concrete specimen (0.5:1:1) with 8 h and 10 MPa pressurization had a density (2.115 g/cm^3^) that was slightly lower than that (2.162 g/cm^3^) of the sand concrete specimen under the same conditions. These results show that the pre-setting pressurization can produce a CDG concrete of similar or even higher values compared with those of the ordinary concrete using sand as an aggregate.

#### 3.1.4. Key Findings

Higher aggregate content and higher water content can increase the density of concrete specimens, while excess water content may reduce the density. The optimum water content was about 22.22% to 25.00% (water–cement ratio = 0.5:1). The 10 MPa pressurization could significantly increase the density values of all the specimens with different mix designs and aggregates, up to 8.31%. The pre-setting pressurization could produce the concrete specimens using CDG soil as an aggregate to obtain similar density values as those of the conventional concrete specimens.

### 3.2. Results of UCS

#### 3.2.1. Effect of Water–Cement–Aggregate Ratio

Generally, the concrete specimens with the water:aggregate:cement ratio of x:1:1.25 had UCS values similar to those with the water:aggregate:cement ratio of x:1:1, where x = 0.4, 0.5, and 0.7, respectively. However, the UCS values of the concrete specimens could increase significantly and then decrease dramatically as the water content increased. As explained in Section 3.1.1, inadequate water content in fresh concrete led to the incomplete hydration reaction of cement, while extra unreacted water increased pores and voids within the concrete. Consequently, these conditions can all result in a decrease in the strength of the hardened concrete. For example, the four CDG specimens (x:1:1.25) with 10 MPa pressurization had UCS values of 27.6, 44.3, 41.0, and 14.1 MPa (average = 31.75 MPa) for x = 0.4, 0.5, and 0.7, respectively. The other four CDG specimens of x:1:1 with 10 MPa pressurization had UCS values of 29.0, 30.4, 44.4, and 13.5 MPa (average = 29.33 MPa) for x = 0.4, 0.5, and 0.7, respectively.

#### 3.2.2. Effect of the Pre-Setting Pressurization

The 10 MPa pressurization could significantly increase the UCS values of the specimens for all six types of mix design. For example, the four CDG specimens (x:1:1.25) with 10 MPa pressurization had an average UCS value of 31.75 MPa. The four CDG specimens (x:1:1.25) without pre-setting pressurization had an average UCS value of 16.20 MPa. The average increase was 95.99%, which is significantly high. For sand concrete specimens with a mix ratio of x:1:1.25, the average UCS values of pressurized specimens and non-pressurized specimens were 32.38 MPa and 23.43 MPa, respectively, and the average increase was 38.21%. On the other hand, CDG concrete and sand concrete specimens hardened under 8 h pressurization exhibited average increases in density values of 152.19% and 73.69%, respectively, which are higher than the average increase. Therefore, it can be illustrated that the 8 h and 24 h pre-setting pressure applications had similar or even better effects on the UCS of specimens according to the results.

#### 3.2.3. Comparisons between CDG and Sand

In general, the CDG concrete specimens had lower UCS values than those of the sand concrete specimens fabricated with 0 MPa pressurization. The CDG concrete (0.5:1:1.25) with 24 h and 10 MPa pressurization had a UCS value (44.3 MPa) that was similar to that (44.9 MPa) of the sand concrete specimen under the same conditions. These results show that the pre-setting pressurization can produce CDG concrete specimens of similar/higher UCS values as those of the sand concrete specimens.

#### 3.2.4. Key Findings

The water content can significantly affect the UCS of the concrete specimens. The optimum water content was about 22.22% to 25.00% (water–cement ratio = 0.5:1). Under 0 MPa pressurization, the CDG concrete specimens had UCS values dramatically lower than those of the sand concrete specimens. The 10 MPa pre-setting pressurization could significantly increase the UCS values of the concrete specimens and produce CDG concrete specimens with similarly high UCS values (44.3 MPa) to those of the sand concrete specimens.

### 3.3. CT Observations

**Figure 11:**
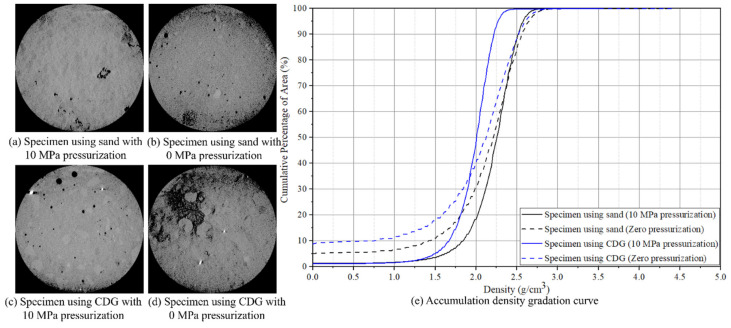
CT images and data analysis of representative specimens: (**a**–**d**) CT images of cross-sections of pressurized and zero-pressurized CDG and sand concrete specimens. (**e**) The accumulation density gradation curves.

The Micro-CT Scanner TOSCANER-3000 (Toshiba, Tokyo, Japan) was used to test the microstructure of CDG and sand concrete specimens with the water:cement:aggregate ratio of 0.7:1:1.25. Slices of the specimens with a width of 10 mm were cut. The four CT images in Figure 11a–d represent the two sand concrete specimens and the two CDG concrete specimens under 10 MPa and 0 MPa pre-setting pressurization, respectively. The gray level of each pixel of the CT images represents the relative density of the pixel area of the CDG or sand concrete specimens. Each gray level is an integer from 0 to 255 and occupies the same pixel area of a specimen cross-section. The darker the gray level of a pixel, the lower the density of the pixel. The void pixel had the 0 gray level. Hence, the gray levels of all the pixels could be used to obtain the density distribution of the specimen slices. The following equations were used to calculate the density level of a pixel according to its gray level:

(3) $$Average\;gray\;level=\frac{\sum_{j=0}^{255}\left(n_{j}\times j\right)}{\sum_{j=0}^{255}n_{j}}$$

(4) $$Density\;of\;the\;i-th\;gray\;level=\frac{Measured\;specimen\;density}{Average\;gray\;level}\times i$$

where *n_j_* = the sub-total number of the pixels with the *j*–th gray level, *j* = 0 to 255. For the four CT images in Figure 11a–d, their measured density values were equal to 2.193, 2.056, 1.958, and 1.898 g/cm^3^, respectively.

The accumulation density gradation curve was calculated using the following equation, accordingly:

(5) $$Accumulative\;density\;gradation\;at\;the\;j-th\;density=\frac{\sum_{j=0}^{i}n_{j}}{\sum_{j=0}^{255}n_{j}}\times 100\%$$

For the four CT images in Figure 11a–d, their accumulative density gradation curves are presented in Figure 11e.

The percentage of the number of void (0 gray level) pixels over the total number of all the pixels for each CT image can be used to represent the porosity of the specimen and can be calculated using Equation (5) at the 0–th density. For the four CT images in Figure 11a–d, their porosity values were, respectively, equal to 1.354%, 5.036%, 1.174%, and 8.718%.

The porosity values of the CDG and sand concrete specimens fabricated with 10 MPa pressurization were 1.174% and 1.354%. They were, respectively, 13.47% and 26.89% of the porosity values 8.718% and 5.036% of the CDG and sand concrete specimens without pre-setting pressurization, respectively. Hence, the 10 MPa pre-setting pressurization can significantly reduce the porosity values.

Secondly, according to Figure 11e, the accumulative percentage areas of less than or equal to the relative density level of 1.5 kg/m^3^ were 3.540% and 4.948% for the CDG and sand concrete specimens fabricated with 10 MPa pressurization. They were significantly lower than the 10.975% and 17.715% obtained for the CDG and sand concrete specimens without pre-setting pressurization.

With zero pre-setting pressurization, the CDG concrete specimen had the porosity of 8.718%, which was higher than that (5.036%) of the sand concrete specimen. With the 10 MPa pressurization, the CDG concrete specimen had the porosity of 1.174%, which was slightly lower than that (1.354%) of the sand concrete specimen. The results showed that the pre-setting pressurization can produce CDG concrete of a similarly lower porosity to that of the sand concrete specimens.

Overall, the two accumulation density gradation curves of the CDG and sand concrete specimens with the 10 MPa pressurization were bonded in zones much narrower than the curves of the two specimens without pre-setting pressurization. This result showed an overall densification of concrete specimens fabricated with 10 MPa pre-setting pressurization.

## 4. Summary and Discussions

In this study, a novel pre-setting pressurization methodology aiming at enhancing the mechanical properties of concrete was developed, and high-quality concrete specimens were fabricated using a self-designed apparatus for applying pre-setting pressure on initial liquid mixtures of fresh concrete. It was illustrated that during the pre-setting pressurization process, the compaction pressure applied on the fresh concrete within the steel cylindrical mold could evenly transmit across diverse constituents of the fresh concrete, including the mortar, aggregates, unreacted free water, and voids.

According to the basic theories of atmospheric pressure, the imposition of static pressure surrounding voids within fresh concrete results in their volumetric reduction. With the application of continuous pre-setting pressure, the diameter of the void decreased, the volume of it contracted, and air entrapped within the void was expelled, thereby leading to a decline in porosity of the pre-setting pressurized concrete specimens.

The reduction in voids provided more space for the movement of aggregates in the fresh concrete matrix, which resulted in denser and more uniform compaction. Meanwhile, the decreased porosity increased the specific surface area of cement mortar, enabling unreacted free water to permeate capillary pores under compaction pressure, thereby facilitating continued hydration reactions. Consequently, the compactness, homogeneity, and compressive strength of the hardened concrete specimens were enhanced.

Additionally, owing to the high water absorption of the clay particles in CDG soil due to their small particle size and large specific surface area, which will reduce the hydration efficiency of cement, the non-pressurized CDG concrete specimens had lower UCS and density values than the non-pressurized sand concrete specimens and exhibited higher porosity. However, under the pre-setting pressurization fabrication, CDG concrete specimens could exhibit comparably high UCS values and low porosity values to those of the sand concrete specimens.

The analysis of experimental results corroborated the mechanism of pre-setting pressurization on properties’ enhancement, as illustrated above. It was demonstrated from the results that the pre-setting pressurization applied on the liquid mixtures of fresh concrete could significantly enhance the characteristics of concrete specimens using different aggregates and with varying mix ratios.

Table 3 summarizes these enhanced properties of concrete specimens fabricated with 10 MPa pre-setting pressurization. By expelling entrapped air and compacting the aggregates and mixture more densely during pressurization, the density of pressurized specimens increased by up to 8.31%, relative to the non-pressurized controls. The density values of pressurized CDG concrete increased by 4.51% on average, comparable to the 4.69% average increase of pressurized sand concrete. Since the hydration reaction in fresh concrete proceeded more completely and cement mortar and aggregate distribution became more uniform under the pre-setting pressurization, the UCS values of pressurized concrete specimens were increased by up to 187.43%. On average, the UCS values of pressurized CDG concrete increased by 91.22%, significantly higher than the 47.58% increase for pressurized sand concrete. According to the image analysis of CT images of pressurized and non-pressurized concrete specimens, it was verified that the pre-setting pressurization reduced the porosity values and enhanced the homogeneity. The porosity of sand concrete specimens was decreased by 73.11%, while the porosity of CDG concrete specimens was decreased by 86.53%. Overall, pre-setting pressurization conferred substantial gains in macroscopic and microstructural properties of concrete specimens.

Various pressurization techniques in previous studies have been investigated and shown to improve the macroscopic and microstructural performance of concrete, including increasing the density value, UCS value, and modulus of elasticity, while lowering water absorption, porosity, and enhancing homogeneity. Different types of pressurization methodologies and improved properties are summarized in Table 4. Compared with these previous methods and corresponding results, it was found that the novel pre-setting pressurization method developed in this study can achieve the same or even better efficiency in improving the mechanical properties of concrete.

## 5. Conclusions

This study aimed to explore the enhancements in the mechanical characteristics, including density, UCS, and porosity, of concrete manufactured under pre-setting pressurization techniques. High-compression pressure was applied on the initial setting of fresh concrete using two types of aggregates, specifically sand and CDG soil. Based on the investigation, the main conclusions can be summarized as follows:

(1) The mix ratio of concrete can affect the density and UCS values of both pressurized and non-pressurized concrete specimens with different types of aggregates. Notably, density and UCS values exhibited significant initial increments followed by sharp decreases as the water content increased. In this study, the optimum water–cement ratio for all types of concrete specimens was 0.5.

(2) The pre-setting pressurization can significantly improve the mechanical properties of both sand concrete and CDG soil concrete. CDG concrete specimens fabricated under 10 MPa pre-setting pressurization exhibited density increments ranging from 2.33% to 6.59%, while the UCS value improvements varied from 32.35% to 171.43%. For sand concrete specimens, the density values were increased by 1.43% to 8.31%, and the UCS values were increased by 8.42% to 187.43%.

(3) The utilization of pre-setting pressurization can effectively expel the excess entrapped air within fresh concrete, thereby reducing the size and quantities of voids. Consequently, the porosity of CDG concrete and sand concrete specimens was decreased by up to 86.53% and 73.11%, respectively. This technique also improved the densification and homogeneity of hardened concrete, leading to an enhancement in their macroscopic mechanical properties.

(4) The application of 10 MPa pre-setting pressurization with a duration of 8 h had an increasing effect on the mechanical properties, similar to those of the 24 h duration pressurization. Specifically, CDG concrete hardened under 8 h pressurization exhibited average increases in density values and UCS values of 6.73% and 152.19%, respectively. In contrast, sand concrete fabricated under the same 8 h pressurization conditions showed average increases in density and UCS of 8.17% and 73.69%, respectively.

(5) Non-pressurized concrete specimens utilizing CDG soil as aggregates had density values and UCS values that were lower than those of the non-pressurized sand concrete specimens. The average UCS value of non-pressurized CDG concrete specimens was 28.97% lower than that of non-pressurized sand concrete specimens. However, the efficiency of 10 MPa pre-setting pressurization on enhancing the mechanical properties of CDG concrete was markedly greater than that observed for sand concrete. The average increase in UCS values for CDG concrete reached 91.22%, significantly higher than the 47.58% increase of sand concrete. Overall, the application of pressurization could produce CDG concrete specimens of similar or higher density and UCS values as those of the sand concrete specimens. This indicated that the CDG soil has the potential to be used as an aggregate in the production of the initially pressurized concrete, with enhanced performance.

The findings of this study proved the potential application of pre-setting pressurization in practical engineering for producing high-performance concrete utilizing CDG soil as an aggregate. In the context of ongoing urbanization and industrialization, there has been a marked increase in demand for concrete materials with good mechanical properties and low cost. The utilization of CDG soil, with its low cost, accessibility, and environmental sustainability, as an alternative aggregate can alleviate the problem of excessive excavation and shortage of sand. Meanwhile, this pressurization technique, characterized by its ease of operation, cost-effectiveness, and high efficiency, presents significant feasibility for the manufacture of CDG concrete with enhanced strength and durability. Consequently, CDG concrete with high performance has great utilization value and promising prospects in the future construction industry. Potential applications include the production of prefabricated concrete components of MiC (Modular Integrated Construction), railway sleepers, and immersed tunnel tubes.

There are still several limitations of this study. For instance, due to mold restrictions, the size of the concrete specimens could not meet the standard requirements for testing some specific mechanical properties of concrete. Meanwhile, due to the compression of fresh concrete during the pre-setting pressurization, the height of the hardened pressurized concrete was lower than that of the non-pressurized concrete, which may have resulted in size effects in the UCS tests. Therefore, future research will aim to investigate additional mechanical properties, including modulus of elasticity, tensile strength, flexural strength, and durability, of concrete specimens produced under varying degrees of pre-setting pressurization. Moreover, the steel mold for fresh concrete will be improved, and the characteristics of pressurized concrete specimens using different types of alternative aggregates and of different sizes and shapes, such as concrete cubes, concrete beams, and concrete columns, will also be examined and evaluated.

## Figures and Tables

**Table 1:** Sieving results of CDG soil and Xiamen sand.

Particle Type	Particle Sizes (mm)	Cumulative Passing Percentage (%)	
		ISO Sand	CDG Soil
Gravel	10	100	100
	6.3	100	99.70
	5	100	98.95
	3.35	100	94.76
Coarse sand	2.36	100	88.10
	2	99.93	87.96
	1.18	93.43	73.99
Medium sand	0.6	43.60	63.26
	0.425	36.75	60.25
	0.3	36.13	57.12
Fine sand	0.212	26.23	55.74
	0.15	10.24	54.57
	0.075	0.23	52.66
Silt	0.063	0.21	52.52
Clay	0.002	0	49.83

**Table 2:** Mix design and density and UCS results of 28 CDG and sand concrete specimens.

Duration of Initial Pressurization (h)	Type of Aggregate	Water/Cement/ Aggregate Ratio	Water Content (%)	Density (g/cm^3^)		UCS (MPa)	
				10 MPa Pressurization	0 MPa Pressurization	10 MPa Pressurization	0 MPa Pressurization
24	Sand	0.4:1:1.25	17.78	2.280	2.187	28.2	25.4
		0.4:1:1	20.00	2.151	2.063	24.0	20.8
	CDG	0.4:1:1.25	17.78	2.075	1.952	27.6	15.4
		0.4:1:1	20.00	1.931	1.887	29.0	17.5
24	Sand	0.5:1:1.25	22.22	2.132	2.102	44.9	29.4
		0.5:1:1	25.00	2.166	2.098	54.9	19.1
	CDG	0.5:1:1.25	22.22	2.167	2.033	44.3	17.6
		0.5:1:1	25.00	2.061	2.003	30.4	11.2
8	Sand	0.5:1:1.25	22.22	2.377	-	46.1	-
		0.5:1:1	25.00	2.162	-	36.4	-
	CDG	0.5:1:1.25	22.22	2.193	-	41.0	-
		0.5:1:1	25.00	2.115	-	44.4	-
24	Sand	0.7:1:1.25	31.11	2.193	2.056	10.3	9.5
		0.7:1:1	35.00	2.060	1.902	11.6	10.5
	CDG	0.7:1:1.25	31.11	1.958	1.898	14.1	9.6
		0.7:1:1	35.00	1.747	1.652	13.5	10.2

**Table 3:** Summary of enhanced characteristics of concrete specimens with 10 MPa pre-setting pressurization.

Type of Aggregates	Water/Cement/Aggregate Ratio	Increase in Density (g/cm^3^)	Increase in UCS (MPa)
CDG	0.4:1:1.25	6.30%	79.22%
	0.4:1:1	2.33%	65.71%
	0.5:1:1.25	6.59%	151.70%
	0.5:1:1	2.90%	171.43%
	0.7:1:1.25	3.16%	46.88%
	0.7:1:1	5.75%	32.35%
Sand	0.4:1:1.25	4.25%	11.02%
	0.4:1:1	4.27%	15.38%
	0.5:1:1.25	1.43%	52.72%
	0.5:1:1	3.24%	187.43%
	0.7:1:1.25	6.66%	8.42%
	0.7:1:1	8.31%	10.48%

**Table 4:** Different pressurization methods and corresponding results in previous studies.

Source	Different Methods	Improved Properties
Roy et al. [7]	a. High temperature of 150 °C and 30 MPa pressurization were applied on fresh concrete. b. 60 MPa pressurization was applied on fresh concrete.	a. Increase the UCS value by up to 88.01%. Increase the homogeneity of hardened concrete specimens. b. Increase the UCS value by up to 17.27%. Increase the homogeneity of hardened concrete specimens.
Ipek et al. [10,11]	Six different (0, 5, 10, 15, 20, and 25 MPa) pressures were applied on fresh reactive powder concrete.	Under 25 MPa pre-setting pressurization, the density value of concrete was increased by 7.79%, the UCS value was increased by 103.68%, and the pore structure was improved.
Nematzadeh et al. [13,14]	Specific pressure was applied on fresh concrete with varying extra water contents (0%, 10%, 20%, and 30% of initial water content by weight) to expel excess water and air.	The UCS value of hardened concrete was increased by up to 185.9%, the modulus of elasticity was increased by up to 100.5%, and the water absorption was decreased by up to 49.7%.
Justs et al. [9]	Five different (10, 20, 30, 40, and 50 MPa) pressures were applied on fresh concrete.	Under 50 MPa pressurization, the density value of hardened concrete was increased by 6.39%, the UCS value was increased by 48%, and the porosity was decreased by 75.64%.
Wang et al. [15,16]	Compaction pressurization was applied on fresh concrete in three layers. Each layer of the concrete was compressed to the designated height under the pressurization.	The UCS value of hardened concrete was increased by up to 100%, and the modulus of elasticity was increased by up to 50%.

## Data Availability

The original contributions presented in the study are included in the article, further inquiries can be directed to the corresponding author.
